# Children with bilateral cerebral palsy use their hip joint to complete a step-up task

**DOI:** 10.3389/fnhum.2024.1343457

**Published:** 2024-02-20

**Authors:** Vatsala Goyal, Keith E. Gordon, Theresa Sukal-Moulton

**Affiliations:** ^1^Department of Biomedical Engineering, McCormick School of Engineering, Northwestern University, Evanston, IL, United States; ^2^Department of Physical Therapy and Human Movement Sciences, Feinberg School of Medicine, Northwestern University, Chicago, IL, United States; ^3^Edward Hines Jr. Veterans Administration Hospital, Hines, IL, United States; ^4^Department of Pediatrics, Feinberg School of Medicine, Northwestern University, Chicago, IL, United States

**Keywords:** pediatrics, stair climbing, biomechanics, cerebral palsy, motor impairments

## Abstract

Performance in stair-climbing is largely associated with disruptions to mobility and community participation in children with cerebral palsy (CP). It is important to understand the nature of motor impairments responsible for making stairs a challenge in children with bilateral CP to clarify underlying causes of impaired mobility. In pediatric clinical populations, sensitive measurements of movement quality can be captured during the initial step of stair ascent. Thus, the purpose of this study was to quantify the lower limb joint moments of children with bilateral CP during the stance phases of a step-up task. Participants performed multiple stepping trials in a university gait laboratory. Outcome measures included extensor support moments (the sum of hip, knee, and ankle sagittal plane moments), hip abduction moments, and their timing. We recruited seven participants per group. We found that peak support and hip abduction moments were similar in the bilateral CP group compared to the typical development (TD) group. We also found that children with bilateral CP timed their peak moments closer together and increasingly depended on the hip joint to complete the task, especially in their more affected (MA) lower limb. Our investigation highlights some underlying causes that may make stair climbing a challenge for the CP population, including a loss of selective voluntary motor control (SVMC), and provides a possible treatment approach to strengthen lower limb muscles.

## 1 Introduction

While the majority of children with bilateral cerebral palsy (CP) are ambulatory (Novak, 2014), stairs and curbs present an exhausting environmental barrier for this population. Performance in stair-climbing is largely associated with disruptions to mobility and community participation in CP, more so than performance in walking (Lepage et al., 1998). Despite this, research investigating movement strategies during stair climbing in bilateral CP has been limited. It is important to understand what makes stair climbing difficult in children with bilateral CP to clarify underlying causes of impaired mobility. This is a major concern because reduced mobility can lead to a higher risk of comorbidities such as heart disease and chronic pain in adulthood (van der Slot et al., 2013; Heyn et al., 2019; Peterson et al., 2020; Schmidt et al., 2020; Hammam et al., 2021). Most importantly, community members affected by CP prioritize research focused on understanding the nature of impairments to improve overall mobility (Vargus-Adams and Martin, 2009, 2011; Gross et al., 2018), especially in the lower limbs (Zvolanek et al., 2022).

In pediatric clinical populations, sensitive measurements of movement quality can be captured during the initial step of stair ascent (Stania et al., 2017). Individuals with and without CP spend approximately 70% of an inclined gait cycle in the stance phase (Ma et al., 2019), suggesting that researchers should prioritize this phase of a step-up task. In individuals with typical development (TD), substantial hip abduction moments and extensor support moments, especially from the knee and ankle, are used to complete a step-up task (Wang and Gillette, 2018; Goyal et al., 2023a,b). However, bilateral lower limb motor impairments from bilateral CP can affect the biomechanics of this task. Specifically, researchers have identified paresis, or weakness, in both the hip abductors and lower limb extensors in bilateral CP (Wiley and Damiano, 1998; Barber et al., 2012; Steele et al., 2012). Adults with stroke, who also experience paresis in the same joint directions, generate lower hip abduction, hip extension, and knee extension moments compared to adults without stroke during a step-up (Goyal et al., 2023a). A reduction in selective voluntary motor control (SVMC) of distal joints such as the knee and ankle is also an often-observed motor impairment in bilateral CP (Sanger et al., 2006; Fowler and Goldberg, 2009; Fowler et al., 2010; Zhou et al., 2017). One group of researchers found that children with CP may compensate for this coordination issue during level-ground walking by shifting kinetic output from the ankle to the hip (Riad et al., 2008). A loss of SVMC may also lead to simultaneous and coupled lower limb movements which alter timing in the gait cycle compared to children without CP (Fowler and Goldberg, 2009). Collectively, this prior research suggests that the stance phase biomechanics of a step-up task may be different in children with bilateral CP compared to children without bilateral CP. Of further importance, understanding the nature of motor impairments in CP can also offer insight into how the central nervous system is working (Sukal et al., 2007; Sukal-Moulton et al., 2013, 2014a,b; Sánchez et al., 2018; Hill and Dewald, 2020) during a challenging activity of daily living.

The purpose of this study was to quantify the lower limb joint moments of children with bilateral CP during the stance phases of a step-up task. We hypothesized that children with bilateral CP would generate lower peak hip abduction and extensor support moments compared to children with typical development (TD), and that timing of these peak moments would occur closer together in children with bilateral CP. We also hypothesized that children with bilateral CP would shift torque generation from the knee/ankle to the hip to successfully complete a step-up task.

## 2 Materials and methods

### 2.1 Participant recruitment

Participants with bilateral CP were recruited through the Shirley Ryan AbilityLab and the Cerebral Palsy Research Registry (Hurley et al., 2011). Inclusion criteria for these individuals were (1) between the age of 5 and 19 years, (2) a medical diagnosis of bilateral CP affecting the lower limbs, (3) Gross Motor Function Classification System (GMFCS) level I-III, and (4) independent ambulatory function with ability to step up with or without assistive devices. Exclusion criteria were (1) botulinum toxin injections to lower limb muscles in the past 6 months, (2) surgeries affecting lower limb function in the past year, and (3) serious comorbidities or cognitive dysfunction that would affect ability to participate. Age and sex-matched participants without bilateral CP (typical development or TD) were recruited through word-of-mouth and flyers. This study was approved by Northwestern University’s Institutional Review Board. Participants under the age of 18 provided assent in addition to informed consent from their parent/guardian, while participants 18 and older provided informed consent themselves.

### 2.2 Set-up and protocol

**FIGURE 1:**
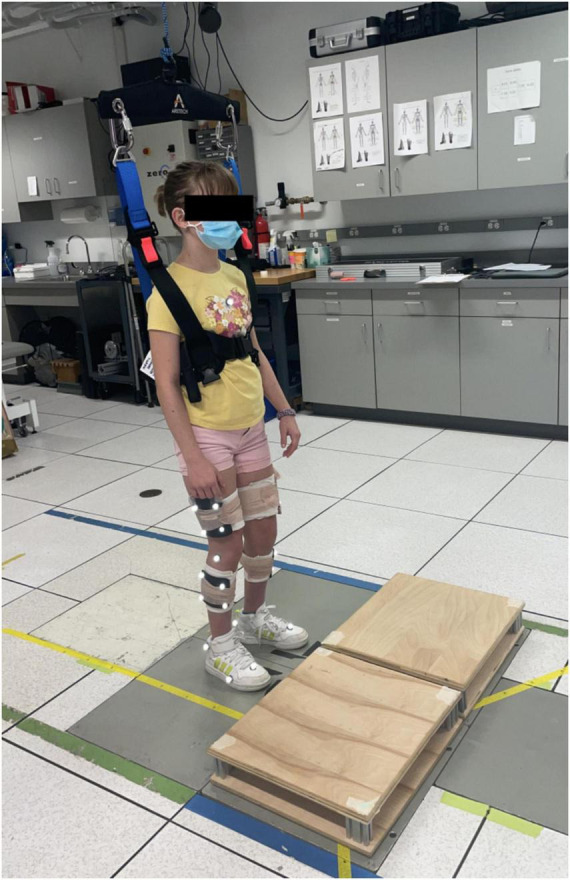
A participant outfitted with reflective markers in the starting position behind the two raised 10.2-cm platforms.

Participants performed multiple stepping trials on a 2 × 2 cluster of force plates (AMTI, Watertown, MA, USA) (Figure 1). Two 10.2-cm wooden platforms, each approximately the size of a single force plate, were placed on two side-by-side force plates to simulate a step. A previous study found that a 10.2-cm step height is both challenging and achievable for clinical populations with lower limb impairments (Goyal et al., 2023a). A 10-camera motion capture system (Qualisys, Göteborg, Sweden) recorded lower limb kinematics from retro-reflective markers placed on the trunk (sternum, C7 vertebrae, T10 vertebrae), pelvis (sacrum, posterior superior iliac spines, greater trochanters), and lower extremities (lateral femoral epicondyles, lateral malleoli, calcanei, the second and fifth metatarsals, and thigh and shank four-marker clusters). Ground reaction forces were captured at 1000 Hz, while kinematics were captured at 100 Hz. Participants also wore a harness that was attached to a passive overhead trolley to minimize the risk of falling.

Participants started the experiment with their feet on two independent force plates posterior to the two platforms. This ensured that all ground reaction forces for the left and right lower limbs were recorded separately. Participants were instructed to step up onto the platform at their typical walking speed (i.e., at the pace if they were walking with a friend, not rushing to catch a bus and not slowly moving in a big crowd). After a short pause on the step, participants were then instructed to step down and backward onto the starting force plates. These step-up trials were repeated 5–15 times per leading foot, depending on participant fatigue and comfort. Once the typical speed was established, we monitored that trials were consistently being completed within 10% of this speed. We offered breaks and asked for participant report of their fatigue. A licensed physical therapist guarded and offered support as needed for safety to participants who were GMFCS level III; we did not offer these participants a handrail or other consistent upper extremity support and they did not use their assistive devices. The trial did not begin until they were standing independently. These participants used a hand touch on the physical therapist’s hand if they felt they needed it. If they attempted to use anything more than support for balance (e.g., holding hands or pulling with arms to ascend), they were cued to reduce this type of support and the trial was not used for analysis. In addition to the step-up trials, all participants completed timed single-limb stance tests (Newton, 1989) and the Waterloo Footedness survey (Elias et al., 1998) to identify the dominant (TD) or less affected (bilateral CP) lower limb. Participants with bilateral CP also completed the Selective Control Assessment of the Lower Extremity (SCALE) (Fowler et al., 2009) and the locomotion ability assessment for kids (ABILOCO-Kids) (Caty et al., 2008).

### 2.3 Data and statistical analysis

Qualisys Track Manager (Qualisys, Göteborg, Sweden) recorded marker and ground reaction force data. Marker data were visually inspected to ensure that they were correctly labeled. All data were then imported to Visual 3D (C-Motion, Germantown, MD, USA). First, a static trial was used to estimate a biomechanical model using the following markers: trunk (sternum, C7 vertebrae, T10 vertebrae, acromions), pelvis (sacrum, posterior superior iliac spines, greater trochanters, ischial tuberosities), thigh (four-marker clusters, greater trochanters, lateral and medial femoral epicondyles), shank (four-marker clusters, lateral and femoral epicondyles, lateral and medial malleoli), and foot (lateral and medial malleoli, calcanei, first, second and fifth metatarsals). Dempster’s regression equations were used to define segment weights (Dempster, 1995) and segment initial properties were estimated based on segment weight and geometry (Hanavan, 1964). Bell’s regression equations were used to define hip joint centers (Bell et al., 1989), while knee and ankle joint centers were estimated based on the static markers defined above. At least three markers from each segment were used to track segment movement. The marker and ground reaction force data were first interpolated to fill in small gaps and passed through a 4th-order low-pass Butterworth filter with a 6 Hz cutoff frequency to filter out high-frequency oscillations. These data were then used in combination to perform inverse dynamics calculations of hip moments in the frontal plane and hip, knee, and ankle moments in the sagittal plane. The joint angular convention used was the Cardan sequence—global axes were used to define overall metrics and local axes were used to define joint angles. To account for the 10.2-cm step height, the height of the two corresponding force plates was adjusted virtually. Important gait events, including lift-off and initial contact for both lower limbs, were identified by the software using a 5 N ground reaction force threshold and manually inspected for accuracy.

Joint moment data were further analyzed in MATLAB (MathWorks, Inc., Natick, MA, USA). All joint moments were normalized to participant body weight for comparison in statistical analyses. Hip, knee, and ankle sagittal plane moments were considered separately and together, summed to calculate an overall extensor support moment (Novak and Brouwer, 2011). All data were evaluated for each individual trial during two stance phases of the step-up. The push-off phase was defined when the trailing limb was in single-limb stance, between leading limb lift-off and initial contact with the step. The pull-up phase was defined when the leading limb was in single-limb stance, between trailing limb lift-off and initial contact with the step. For each individual participant, any step-up trial with a length outside of two standard deviations from the average were not considered in statistical analysis.

All statistical analyses were performed in Stata IC 14.1 (StataCorp LLC, College Station, TX, USA) and the threshold for significance was set to *p* < 0.05. Independent two-sample *t*-tests were used to compare participant-specific metrics such as age, weight, and height. An ANOVA was used to compare single limb stance times between the limbs in each group [CP more affected (MA), CP less affected (LA), typical development dominant (TD)]. Paired *t*-tests were also used to compare SCALE scores between the limbs of participants with bilateral CP. All outcome measures were considered independently for each stance phase: (1) peak hip abduction moments, (2) peak support moments, (3) individual hip, knee, and ankle percent contributions to peak support moments, (4) time duration of stance phase, and (5) time to peak moments. Individual joint percent contributions were calculated by dividing hip, knee, and ankle contributions to peak support moment by the overall peak support moment. Timing of peak moments was identified as a percentage of the corresponding stance phase. Our analysis determined that the effect of limb dominance was not significant for children with TD (Goyal et al., 2023b); as such, only data from their dominant limb was considered in subsequent analyses. All outcome measures were statistically compared using linear mixed effects models with one fixed effect of limb (MA, LA, TD) and a random effect of participant. The distribution of residuals for each mixed effects model was visually inspected using histograms to confirm normality of the data. All trials for each participant were input individually into the statistical models to increase the effective sample size. Significance of multiple pairwise comparisons was adjusted using Bonferroni’s corrections. Finally, the effect size of each statistical analysis was calculated using G*Power (Faul et al., 2009).

## 3 Results

### 3.1 Participant summary

We recruited 7 participants in each group (Table 1). There were no significant differences in age, weight, and height between the groups. Five participants with bilateral CP were GMFCS level II and two participants were GMFCS level III. No participants with CP had notable joint contractures in the lower extremities that influenced their ability to perform the step up task. Single limb stance times between the limbs were significantly different (*p* = 0.002), where the TD limb had a larger stance time than the LA (*p* = 0.007) and MA limbs (*p* = 0.004). Average SCALE scores were significantly different between the limbs of the CP group (*p* = 0.020).

**TABLE 1:** Mean (SD) participant-specific metrics and clinical assessment outcomes.

Outcome measure	Group		
	Bilateral CP (*n* = 7)		TD (*n* = 7)
Age (years)	10.5 (3.6)		10.2 (3.9)
Limb dominance	2R/5L		7R
Weight (kg)	38.7 (19.6)		41.7 (23.7)
Height (m)	1.40 (0.19)		1.42 (0.22)
ABILOCO-Kids	14.1 (5.7)		–
**Limb**	**MA**	**LA**	**Dom**
Single-limb stance time (s)	2.27 (2.28)	5.25 (5.91)	43.7 (38.2)
SCALE	3.15 (1.68)	6.14 (3.58)	–

### 3.2 Push-off stance phase

**FIGURE 2:**
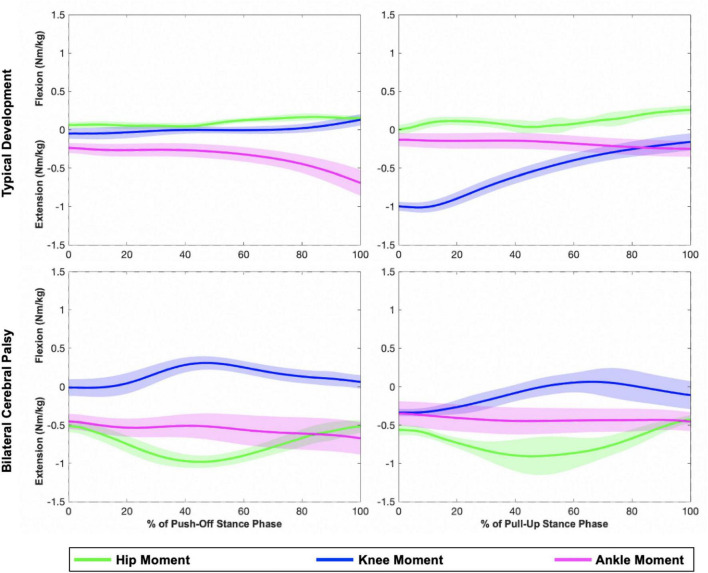
Representative plots of hip, knee, and ankle sagittal plane moments during the push-off (**left**) and pull-up (**right**) stance phases of a step-up task. The **top** row displays joint moments from an individual in the TD group, while the **bottom** row displays joint moments from an individual in the bilateral CP group (MA limb). Shaded regions represent one standard deviation. Compared to the individual with TD, the individual with CP generated larger hip extension moments.

**FIGURE 3:**
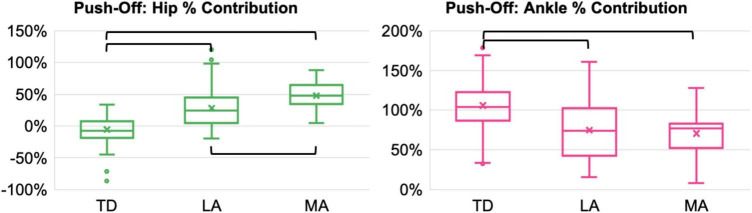
Hip and ankle percent contributions during the push-off stance phase of a step-up task for each lower limb (TD = typical development, LA = bilateral CP less affected, MA = bilateral CP more affected). Significant pairwise comparisons are shown by the black brackets (corrected *p* < 0.017).

There were no significant differences in peak hip abduction or support moments between the limbs in the push-off stance phase (Table 2). There were significant differences in hip (*p* < 0.001, effect size = 0.943) and ankle (*p* = 0.011, effect size = 0.492) percent contributions to peak support moments between the limbs (Figures 2, 3). The MA limb had a significantly higher hip percent contribution compared to the LA and TD limbs, while the LA limb also had a significantly higher hip percent contribution compared to the TD limb (*p* < 0.001 for all). In contrast, both limbs of participants with CP had lower ankle percent contributions compared to the TD limb (less affected: *p* = 0.016; more affected: *p* = 0.003). Joint moments in Nm/kg for the hip, knee, and ankle at the time of peak support moments are provided in Table 2, though statistics were not run on these values.

**TABLE 2:** Mean (SD) outcome measures for the push-off and pull-up stance phases in a step-up task.

Metric	Push-off stance phase			Pull-up stance phase		
	**Bilateral CP**		**TD**	**Bilateral CP**		**TD**
	**More affected**	**Less affected**	**Dominant**	**More affected**	**Less affected**	**Dominant**
**Joint moment (Nm/kg)**						
Peak hip abduction	0.598 (0.206)	0.552 (0.188)	0.658 (0.178)	0.553 (0.264)	0.573 (0.214)	0.520 (0.192)
Peak support	−1.08 (0.227)	−1.08 (0.321)	−0.887 (0.333)	−1.55 (0.219)*	−1.66 (0.405)*	−1.23 (0.180)
**Individual joint moments at the time of peak support moments (Nm/kg)**						
Hip	−0.531 (0.257)	−0.270 (0.255)	0.015 (0.143)	−0.726 (0.189)	−0.501 (0.183)	−0.292 (0.226)
Knee	0.201 (0.241)	−0.046 (0.387)	0.017 (0.261)	−0.352 (0.260)	−0.666 (0.246)	−0.657 (0.253)
Ankle	−0.751 (0.288)	−0.765 (0.359)	−0.873 (0.278)	−0.469 (0.216)	−0.494 (0.230)	−0.225 (0.184)
**Individual percent contributions to peak support moments (%)**						
Hip	48.0 (20.3)*^+^	28.4 (28.7)*	−5.86 (20.9)	47.2 (11.4)*^+^	31.5 (17.0)	20.2 (17.0)
Knee	−18.6 (22.3)	−2.96 (42.9)	0.017 (33.7)	22.3 (16.0)*^+^	39.4 (13.4)*	60.3 (19.6)
Ankle	70.6 (27.8)*	74.6 (37.4)*	106 (30.8)	30.6 (14.3)	29.1 (12.8)	19.5 (14.9)
**Temporal**						
Average stance time (s)	0.533 (0.107)^+^	0.604 (0.155)	0.513 (0.092)	0.479 (0.106)	0.539 (0.240)	0.470 (0.083)
Time of peak hip abduction (% of stance phase)	22.6 (0.092)*	25.4 (0.124)*	16.4 (0.089)	51.9 (22.5)	42.4 (23.3)	45.3 (31.3)
Time of peak support (% of stance phase)	57.6 (36.4)*	57.8 (36.2)*	94.1 (0.181)	17.1 (15.2)*^+^	10.4 (7.15)	7.03 (4.70)

For individual percent contributions to peak support moment, a negative percentage indicates a joint flexion contribution.

*p < 0.05 for a significantly different from control limb.

^+^*p* < 0.05 for a significantly different from less affected limb.

There were significant differences in average stance time of the push-off phase (*p* = 0.005, effect size 0.322), where the LA limb spent more time in the stance phase than the MA limb (*p* = 0.001) (Table 2). There were also significant differences in timing of peak moments between the limbs (both *p* < 0.001, effect size = 36.5 for peak hip abduction moment and 0.580 for peak support moment). Both limbs of participants with CP reached a peak hip abduction moment later compared to the control limb (LA: *p* < 0.001; MA: *p* = 0.004) and reached a peak support moment earlier compared to the TD limb (LA: *p* < 0.001; MA: *p* < 0.001).

### 3.3 Pull-up stance phase

**FIGURE 4:**
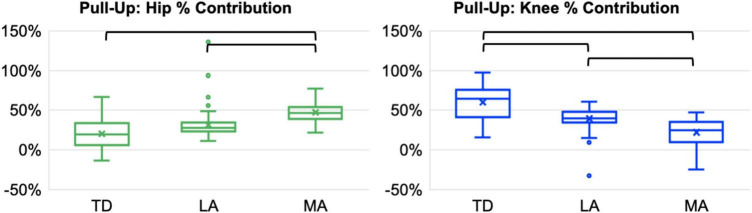
Hip and knee percent contributions during the pull-up stance phase of a step-up task for each lower limb (TD = typical development, LA = bilateral CP less affected, MA = bilateral CP more affected). Significant pairwise comparisons are shown by the black brackets (corrected *p* < 0.017).

There were significant differences in peak support moments (*p* < 0.001, effect size = 0.640) between the limbs in the pull-up stance phase (Table 2). Both the limbs of participants with CP generated higher peak support moments compared to the TD limb (LA: *p* = 0.016; MA: *p* = 0.001). There were also significant differences in hip and knee percent contributions to peak support moments between the limbs (both *p* < 0.001, effect size = 0.719 for hip and 0.942 for knee) (Figures 2, 4). The MA limb had a significantly higher hip percent contribution compared to the LA and TD limbs (both *p* < 0.001). In contrast, the MA limb had a significantly lower knee percent contribution compared to the LA and TD limbs (both *p* < 0.001), while the LA limb also had a significantly lower knee percent contribution compared to the TD limb (*p* = 0.003). Joint moments in Nm/kg for the hip, knee, and ankle at the time of peak support moments are provided in Table 2, though statistics were not run on these values.

There were no significant differences in the average stance time of the pull-up phase between the limbs, though there was a significant difference in time of peak support moment (*p* = 0.042, effect size = 0.419) (Table 2). The MA limb reached a peak support moment later compared to the LA (*p* = 0.020) and TD limbs (*p* = 0.012).

## 4 Discussion

The present study quantified differences in the lower limb joint moment strategies of a step-up task between children with and without bilateral CP. While there were only small differences in peak moments between the CP and TD groups, there were significant differences in timing of these moments during each stance phase of a step-up task. We also quantified an increased dependence on the hip joint to keep the body upright during a step-up with an associated decreased use of the knee and ankle joints in the CP group, especially in the MA limb. These results can further help us narrow down target areas to improve movement quality in bilateral CP.

Our first hypothesis that peak hip abduction and extensor support moments of a step-up task would be lower in children with bilateral CP was not confirmed. Given that step initiation requires larger frontal plane moments compared to subsequent steps (Wang and Gillette, 2018), our results suggest that a step-up task can be used as a hip abductor strengthening activity in individuals with CP because it provides a functional load to the hip abductor muscles. Children with bilateral CP unexpectedly generated significantly larger peak support moments in the pull-up phase compared to controls, reflective of the high demand from the lower limb extensors required by this phase of stair ascent (Riener et al., 2002; Reeves et al., 2009; Novak and Brouwer, 2011; Strutzenberger et al., 2011). Despite extensor muscle weakness in the hip, knee, and ankle (Wiley and Damiano, 1998), children with CP in our study were successful in completing the step-up task, meaning that they were able to meet the minimum support moment threshold. However, the greater effort output by the extensors during the pull-up phase may be part of an alternative and inefficient strategy to step up, similar to that which has been quantified in other clinical populations and older adults (Reeves et al., 2009; Brown et al., 2016).

**FIGURE 5:**
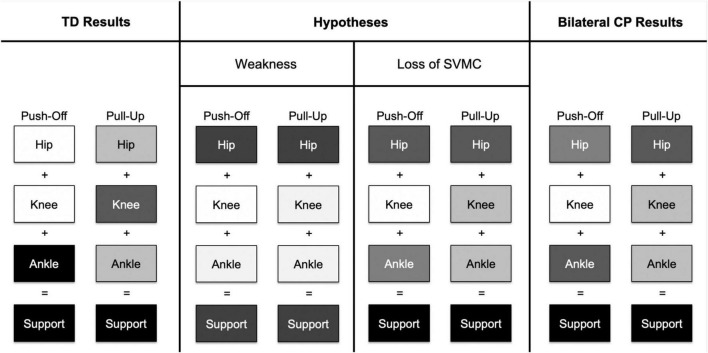
This figure depicts a concept map of contributions to extensor support moments from the hip, knee, and ankle joints. A darker shade of an individual joint indicates a larger contribution to support moment. In the TD group, the ankle joint had the largest contribution to support moments in the push-off phase while the knee had the largest contribution to support moments in the pull-up phase. If distal joint weakness was the primary impairment affecting gait in children with bilateral CP, we hypothesize that the hip joint compensates for low knee and ankle joint contributions in both stance phases. We might also hypothesize that overall support moments would be lower in the bilateral CP group compared to the TD group. However, if a loss of SVMC was the primary impairment affecting gait, we hypothesize that (1) hip contribution increases as compensation for decreased contribution from the ankle joint only in the push-off phase and (2) hip contribution increases as compensation from decreased contribution from the knee joint only in the pull-up phase. Indeed, the results from the bilateral CP group point toward a loss of SVMC, as there was a pattern change between the two different stance phases rather than an overall decrease in contributions from both the knee and ankle joints in both stance phases.

Indeed, children with bilateral CP used their hip extensors more and their knee and ankle extensors less compared to controls when stepping up, which confirms our second hypothesis. This strategy is especially prevalent in the MA limb. Previous research has quantified a similar shift in children with hemiparetic CP during gait (Riad et al., 2008) and adults with hemiparetic stroke during stair ascent (Goyal et al., 2023a), possibly as a compensation for distal weakness in the paretic limb. However, weakness may not be the only impairment affecting the distal joints in children with bilateral CP. Recall that in the TD group, the ankle contributes the most to support moments in the push-off phase while the knee contributes the most to support moments in the pull-up phase. In the CP group, while the ankle joint did not produce equally enough plantarflexion during the push-off phase (Table 2), it did during the pull-up phase of a step-up task. This change in pattern and capacity between two different contexts, which in this case are the two stance phases, suggests that a loss of SVMC in the distal joints (Sanger et al., 2006) may primarily be responsible for the shift in hip dependence during a step up (Figure 5).

The ability to independently activate the joints is significantly reduced in the knee and ankle compared to the hip (Fowler et al., 2010) and has been correlated with abnormal gait patterns in CP (Chruscikowski et al., 2017; Zhou et al., 2019). Researchers have hypothesized that a loss of SVMC is due to corticospinal tract damage and compensatory use of brainstem motor pathways, including the rubrospinal, reticulospinal, and vestibulospinal tracts (Fowler et al., 2010; Cahill-Rowley and Rose, 2014; Zhou et al., 2017; Sánchez et al., 2018). In general, the brainstem motor pathways have connections to the hip joint for postural control. Stimulations to activate the human vestibular system have induced activity in hip extensor muscles such as the gluteus medius and biceps femoris (Ali et al., 2003), which might explain the notable hip extension activity in the CP group during a step-up task. Future interventions to improve mobility in CP may benefit from focusing on strengthening the hip joint (Riad et al., 2008) to optimize its function as compensation for distal joint impairment. In addition, a focus on improving distal SVMC ability during early intervention in bilateral CP (Riad et al., 2008; Sargent et al., 2020) may improve efficiency of their mobility and limit the need for dependence on the hip joint during gait and stairs.

Increased dependence on the hip extensors may also explain why timing of peak hip abduction moments and support moments were closer together in both limbs of participants with CP compared to the TD limb. During stair ascent, the gluteus maximus muscle plays a role in both hip extension and hip abduction (Lyons et al., 1983). We postulate that this muscle played a larger role in contributing to overall extension in our participants with bilateral CP, and therefore influenced the timing of both peak moments. In addition to closer peak moments, timing of the first peak moment in each stance phase occurred later in the CP limbs compared to the control limb. As the rate of force development is significantly lower in children with bilateral CP compared to children with TD during isometric conditions (Moreau et al., 2012), it may be inferred that the ability to rapidly generate torque in dynamic conditions is also impeded. This delay also suggests an increased use of brainstem motor pathways, as multiple synapses increases the central motor conduction time compared to the corticospinal pathways (Eyre et al., 2001; Lemon, 2008).

In this study, we quantified the differences in lower limb joint moments between children with and without bilateral CP during a functional step-up task. Limitations of the study include instructing participants to move at a self-selected speed, which was mitigated through normalization of the stance phases, and the low sample size in each group. Our investigation highlights some underlying causes that make stairs a challenge for the CP population and provides a possible treatment approach to strengthen lower limb muscles. To replicate the type of step ups quantified in our study, patients should perform stairs with as little external support (from a person or railing) as is feasible and safe so they can overload the lower extremity muscles. If significant compensations through the trunk or lower extremity kinematics occurs, strategies that unload body weight may be indicated to maintain appropriate alignment. Rehabilitation focused on optimizing use of the hip extensors and improving distal joint selective control may lead to better outcomes for children with bilateral CP.

## Data availability statement

The raw data supporting the conclusions of this article will be made available by the authors, without undue reservation.

## Ethics statement

The studies involving humans were approved by the Northwestern University Institutional Review Board. The studies were conducted in accordance with the local legislation and institutional requirements. Written informed consent for participation in this study was provided by the participants’ legal guardians/next of kin if they were under the age of 18 years and the participants themselves if they were 18 years of age or older.

## Author contributions

VG: Conceptualization, Data curation, Formal analysis, Funding acquisition, Investigation, Methodology, Project administration, Validation, Visualization, Writing – original draft, Writing – review and editing. KG: Data curation, Funding acquisition, Methodology, Resources, Supervision, Writing – review and editing. TS-M: Conceptualization, Funding acquisition, Investigation, Methodology, Project administration, Supervision, Validation, Writing – review and editing.
